# Human Breast Milk Promotes the Secretion of Potentially Beneficial Metabolites by Probiotic *Lactobacillus reuteri* DSM 17938

**DOI:** 10.3390/nu11071548

**Published:** 2019-07-09

**Authors:** Tu T. Mai, Dat Q. Tran, Stefan Roos, J. Marc Rhoads, Yuying Liu

**Affiliations:** 1 Department of Pediatrics, Division of Gastroenterology, The University of Texas Health Science Center at Houston McGovern Medical School, Houston, TX 77030, USA; 2 Department of Microbiology, Uppsala BioCenter, Swedish University of Agricultural Sciences, 75007 Uppsala, Sweden

**Keywords:** probiotic, human breast milk, formula, inflammation, metabolite

## Abstract

Human breast milk (HBM) may have beneficial effects on *Lactobacillus reuteri* DSM 17938 (LR 17938) -mediated immunomodulation. We aimed to determine the effects of HBM on proliferation of LR 17938 in vitro and its associated proteins and metabolites in culture, in order to provide mechanistic insights into the health benefits of LR 17938. LR 17938 was cultured anaerobically in MRS bacterial culture media, HBM (from 6 mothers), and 2 types of cow-milk formula. The colony-forming unit (CFU) was calculated to evaluate LR 17938 growth. Sixteen-hour-fermented supernatants were used for metabolomics, and bacterial lysates were used for proteomics analysis. We found that growth of LR 17938 was 10 times better in HBM than in formula. We detected 261/452 metabolites upregulated when LR 17938 cultured in HBM compared to in formula, mainly participating in the glyoxylate cycle (succinate), urea cycle (citrulline), methionine methylation (N-acetylcysteine), and polyamine synthesis (spermidine). The significantly up-regulated enzymes were also involved in the formation of acetyl-CoA in the glyoxylate cycle and the antioxidant N-acetylcysteine. In conclusion, HBM enhances the growth of LR 17938 compared to formula and promotes LR 17938-associated metabolites that relate to energy and antioxidant status, which may be linked to the physiological effects of *L. reuteri*.

## 1. Introduction

Clinical studies have shown beneficial effects of probiotics in preventing and/or treating various gastrointestinal diseases, including necrotizing enterocolitis, infantile colic, irritable bowel syndrome, inflammatory bowel disease, and acute infectious and antibiotic-associated diarrhea [1,2,3,4,5,6]. 

*Lactobacillus reuteri* DSM 17938 (LR 17938) is a probiotic strain derived from *Lactobacillus reuteri* ATCC 55730. The latter strain was originally isolated from a Peruvian mother’s breast milk but was then found to carry two plasmid-borne antibiotic resistance genes, which were then removed to create the daughter strain LR 17938 [7]. LR 17938 has been shown to inhibit pathogen growth, modulate the immune system, and exert strong anti-inflammatory effects when fed to newborn rats and mice [8,9,10]. Feeding LR 17938 to newborn dam-fed rats with necrotizing enterocolitis increased the percentage of Foxp3+ regulatory T cells (Tregs) in the ileum [9]. Foxp3+ Tregs are anti-inflammatory cells that induce self-tolerance and maintain intestinal homeostasis. Such an effect was not observed in newborn formula-fed rats, suggesting that factors in breast milk may enhance the immunomodulatory effects of LR 17938 [9]. An increase in Tregs and tolerogenic dendritic cells (DCs) was observed when newborn rats were fed with LR 17938 and human breast milk (HBM) vs LR 17938 and formula [11]. 

Various factors in human breast milk play an important role in modulating the immune system, including IgA, lactoferrin, β-lactoglobulin and α-lactoglobulin, epidermal growth factor, and live bacteria (up to 10^9^ CFU per ml), among those lactobacilli and bifidobacteria [12,13,14,15,16]. We suggested that these factors in breast milk promote the immune modulatory functions of LR 17938. Human breast milk also contains bioactive components, among those human milk oligosaccharides (HMO), which mediate immune responses through different mechanisms [17]. 2′-Fucosyllactose is the most abundant HMO in most mothers’ breast milk; and it has now been synthesized and supplemented in some infant formulas, yielding immune benefits and decreasing respiratory infections [18,19]. It is known that LR 17938 produces biologically active metabolites as the probiotic grows in culture. However, it is currently unknown how human breast milk affects the production of these biological products when administered together with LR 17938.

We first studied whether LR 17938 would grow better in human breast milk (HBM) versus formula. LR growth in deMan-Rogosa-Sharpe (MRS, an optimal growth media for lactobacillus), as well as HBM plus casamino acids. A previous study showed that casamino acids, a mixture of amino acids and some small peptides obtained from hydrolyzed casein, can increase the growth of LR 17938 [20]. 

These findings may lead to the identification of a secreted factor (s) or biochemical (s) central to the mechanism of LR 17938 modulation of immune function and may further aid in the discovery of new therapeutic treatments for autoimmune disorders. 

## 2. Materials and Methods 

Probiotic LR 17938 preparation. The probiotic strain LR 17938 was provided by Biogaia AB (Stockholm, Sweden). 

Collection of human breast milk and formula. Human breast milk was collected from six different healthy mothers (of healthy infants aged 4 weeks–7 months). Mothers were instructed to pump and collect samples at home, as they routinely do to collect breast milk for their infants. We collected 60 to 120 mL per mother, depending on availability, as not to interfere with their supply for their own infants. Samples were then stored at 4 °C in their own refrigerator until being collected (within the same day). Samples were brought back to our lab, aliquoted and stored in −20 °C until being used for experiments. Three infant formulas were being used in our experiments (2 regular formula (Similac Advance, Enfamil) and 1 HMO-supplemented formula (Similac Pro-Advance)), either premixed or mixed as directed on the bottle. Only 2 regular formulas (without HMOs) were included in metabolomics studies and proteomics studies.

Quantifying LR 17938 growth in different media. LR 17938 was anaerobically cultured in deMan-Rogosa-Sharpe (MRS; Difco, Detroit, MI, USA) medium, human breast milk, formula and human breast milk plus casamino acids at 37 °C for 24 h. Media (MRS, human breast milk, and formula) without LR were also anaerobically cultured. Culture experiments were performed under 12 different situations. Samples were obtained at 16 h, then plated on MRS agar at 10^6^ serial dilutions and grown anaerobically over 48 h. Quantitative analysis of bacteria was performed by counting colony-forming units (CFU)/mL on MRS agar. 

Evaluation of LR growth in fatty acid. LR 17938 was anaerobically cultured in 20% MRS medium (in combination with (Dulbeco’s Phosphate Buffered Saline 1× (DPBS) medium). Fatty acid supplement was obtained from Sigma-Aldrich (which contained linoleic, oleic, myristic, lauric, and arachidonic acid, with concentrations of each less than 3g/L). Concentration of fatty acid (0.025%, 0.05%, and 0.1%) was used based on suggested amount from Sigma-Aldrich. Samples were obtained at 16 h, then plated on MRS agar at 106 serial dilutions and grown anaerobically over 48–72 h. Quantitative analysis of bacteria was performed by counting colony-forming units (CFU)/mL on MRS agar. 

Metabolomic studies. Global metabolomics were analyzed by Metabolon, Inc (Morrisville, NC, USA). Using mass spectrometry, and the fermented supernatants of the 16-h samples (not including the HMO formula), as we demonstrated that most optimal growth occurred at this point.

### Proteomic Studies 

Sample preparation. Sixteen-hour samples (Formula + LR (*n* = 3), HBM + LR (*n* = 6)) were collected and washed with PBS three times before pellets were stored in −80 °C until ready for use. Only Similac Advance was used as formula for proteomic studies, as samples from Enfamil did not yield adequate protein concentrations for the study. Samples were then resuspended in 500 uL of 40 mM Tris, 30 mM NaCl, pH 8.0, and protease inhibitor. Samples were sonicated 6 times (10 s/cycle, 15 s in between cycles). All samples were then centrifuged at 15.4 g for 5 min at 10 °C, and supernatants were saved. Protein concentrations of each samples were measured using DC™ Protein Assay (BioRad, Hercules, CA, USA) according to manufacturer’s protocol. Aliquots of 50 ug of samples were sent to the University of Texas. Proteomics Center (Houston, TX, USA) for proteomics analysis. Bacterial lysates of Enfamil were not collected due to inadequate protein concentrations. At the University of Texas Proteomics Center, samples were subjected to acetone precipitation; proteins were precipitated at −20 °C for three hours. After centrifugation (12,000 *g* × 5 min), the pellets were resuspended in 10 mL of 150 mM Tris-HCl, pH 8.0, denatured and reduced with 20 mL of 9 M urea, 30 mM DTT in 150 mM Tris HCl, pH 8.0, at 37 °C for 40 min, then alkylated with 40 mM iodacetamide in the dark for 30 min. The reaction mixture was diluted 10-fold using 50 mM Tris-HCl pH 8.0 prior to overnight digestion at 37 °C with trypsin (1:20 enzyme to substrate ratio). Digestions were terminated with adding equal volume of 2% formic acid, and then desalted using Waters Oasis HLB 1 mL reverse phase cartridges according to the vendor’s procedure: wash cartridge with 2 × 500 uL of 70 % of acetonitrile in 0.1% formic acid, equilibrate with 2 × 500 uL of 0.1% formic acid, load total volume of digest, wash with 2 × 500 uL of 0.1% formic acid, and elute with 500 uL of 70% acetonitrile in 0.1% formic acid. Eluates were dried via vacuum centrifugation. 

Sample analysis. About 1 µg of the tryptic digest (in 2 % acetonitrile/solvent A) was analyzed by LC/MS/MS on an Orbitrap FusionTM TribridTM mass spectrometer (Thermo Scientific^TM^, Waltham, MA, USA) interfaced with a Dionex UltiMate 3000 Binary RSLCnano System. Peptides were separated onto a AcclaimTM PepMap TM C18 column (75 mm ID × 15 cm, 2 mm) at flow rate of 300 nl/min. Gradient conditions were: 3%–22% solvent B (B) for 120 min; 22%–35% B for 10 min; 35%–90% B for 10 min; 90% B held for 10 min, (solvent A, 0.1% formic acid in water; solvent B, 0.1% formic acid in acetonitrile). The peptides were analyzed using data-dependent acquisition method, Orbitrap Fusion was operated with measurement of FTMS1 at resolutions 120,000 FWHM, scan range 350–1500 m/z, AGC target 2E5, and maximum injection time of 50 ms; During a maximum 3 s cycle time, the ITMS2 spectra were collected at rapid scan rate mode, with CID NCE 35, 1.6 m/z isolation window, AGC target 1E4, maximum injection time of 35 ms, and dynamic exclusion was employed for 60 s. 

Data processing and analysis. The raw data files were processed using Thermo Scientific^TM^ Proteome Discoverer^TM^ software version 1.4 (Waltham, MA, USA). Spectra were searched against the *Lactobacillus reuteri* strain ATCC 55730/SD2112 database using Sequest ^HT^ search engine. Search results were trimmed to a 1% FDR using Percolator. For the trypsin, up to two missed cleavages were allowed. MS tolerance was set 10 ppm; MS/MS tolerance 0.8 Da. Carbamidomethylation on cysteine residues was used as fixed modification; oxidation of methionine as well as phosphorylation of serine, threonine and tyrosine was set as variable modifications. 

Statistics. Experimental results are expressed as means ± SE. Statistical analysis was performed using one-way ANOVA and correlation (Graph Pad Prism 4.0; GraphPad Software, San Diego, CA, USA). A *p* value < 0.05 was considered statistically significant. Metabolomic data was analyzed by Metabolon, Inc. The raw proteomic data and proteomic data was analyzed by the University of Texas Proteomics Center.

## 3. Results

### 3.1. LR 17938 Growth was Higher in HBM as Compared to Its Growth in Formula 

**Figure 1:**
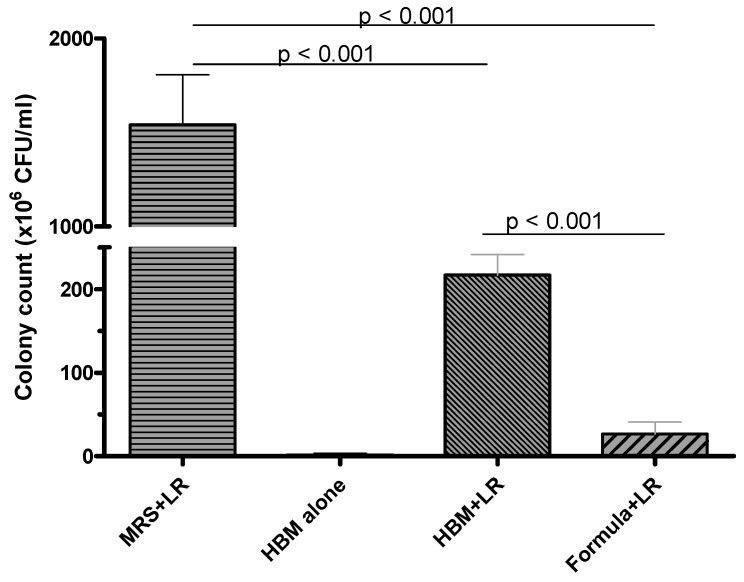
Comparison of *Lactobacillus reuteri* DSM 17938 (LR 17938, or LR) growth in MRS media, human breast milk (HBM), and 2 regular formulas. LR growth was significantly better in HBM (216.7 ± 60.88 × 10^6^ CFU/mL) compared to formula (26.67 ± 35.02 × 10^6^ CFU/mL) (*p* < 0.001).

LR 17938 growth at 16 h was significantly higher (1541 ± 597.4 × 10^6^ CFU/mL) in MRS media, which is a media designed specifically to promote growth of lactobacilli. While comparing its growth in HBM versus 2 regular formulas (Enfamil and Similac Advance), its growth was almost 10-fold higher in HBM (216.7 ± 60.88 × 10^6^ CFU/mL) compared to formula (26.67 ± 35.02 × 10^6^ CFU/mL) (*p* < 0.001) (Figure 1). No LR 17938 was able to grow in MRS or formula alone, and insignificant LR 17938 growth was observed in HBM alone. We found that 1 % casamino acids further promoted LR 17938 growth in HBM (*p* < 0.001) (Supplemental Figure S1).

LR 17938 growth in HMO formula was not significantly better as compared to its growth in regular formulas (Supplemental Figure S2), hence HMO formula with and without LR 17938 were excluded from further studies, including metabolomics and proteomics study.

### 3.2. Metabolomics Data

Using mass spectrometry, 679 metabolites were identified from the culture supernatants. 

**Figure 2:**
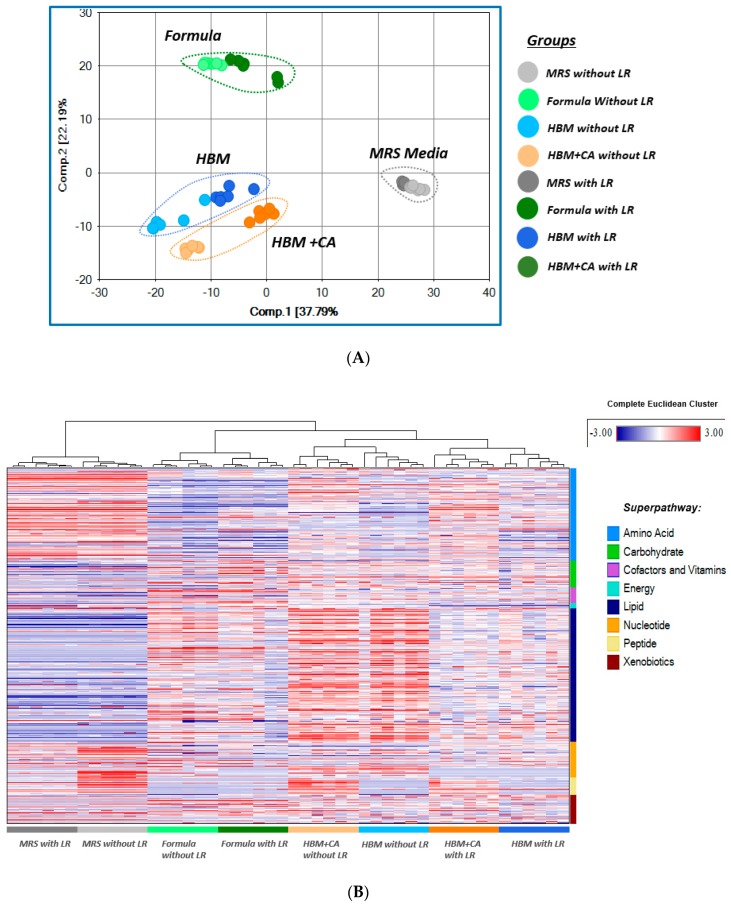
(**A**) Principal component analysis (PCA) showed major segregation among the different media. (**B**) Hierarchical clustering analysis showed major segregation among the groups, based on both media and LR growth status. Each column represents a separate culturing sample (6 samples per group). Each horizontal line represents an identified metabolite (679 metabolites identified from mass spectrometry). Red indicates up-regulated metabolites, while blue indicates down-regulated metabolites.

Both principal components analysis (PCA) and hierarchical clustering analysis (HCA) showed major segregation among metabolites identified in MRS media, HBM, HBM + CA, and formula, with and without LR 17938. Findings suggested that the metabolic differences between the groups were driven mainly by the biochemical composition of the culture media (Figure 2A,B). The addition of LR 17938 in each group of culture media produced some convergence among the groups, suggesting an increased similarity in biochemical composition among the groups with the addition of LR 17938 (Figure 2A). 

In comparison to PCA, hierarchical clustering analysis (HCA) provided a more detailed visual overview of what metabolites are changing in each culturing media. On the heat map, upregulated metabolites are shown in red, and downregulated metabolites in blue. In HBM or HBM + CA without LR 17938, there was a significantly higher abundance of many lipids, as compared to either MRS or formula sample without LR 17938. When compared to LR 17938 growth in formula, LR 17938 growth in HBM or HBM + CA produced a higher increase in amino acid derivatives and more dramatic decrease in lipids (Figure 2B). 

**Figure 3:**
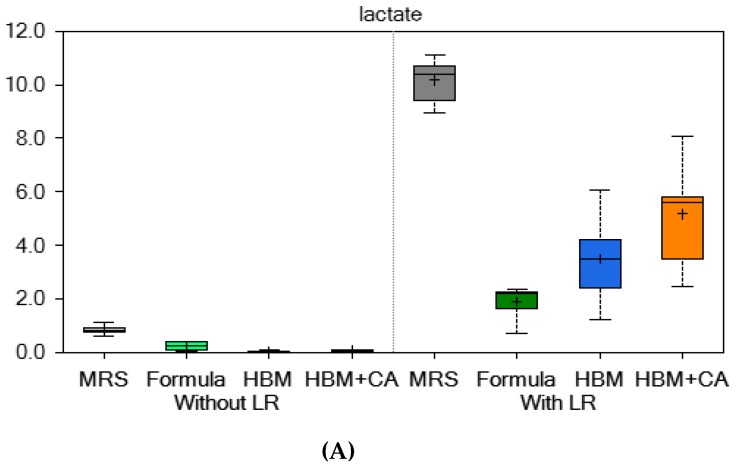

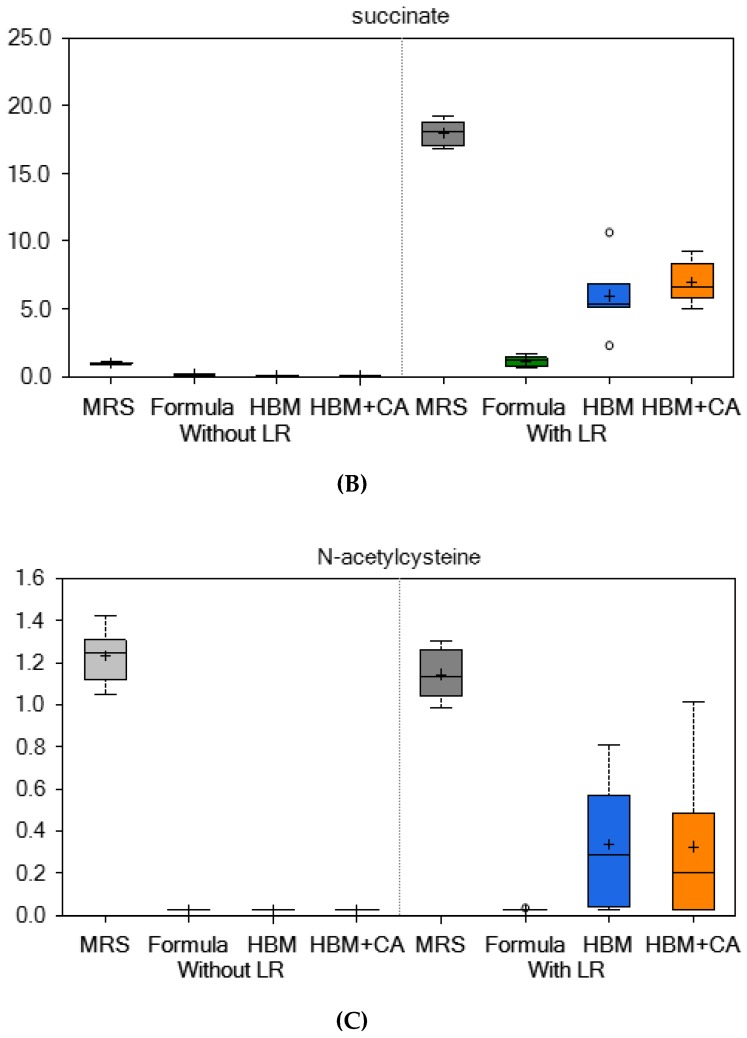
(**A**) Lactate, (**B**) succinate, and (**C**) N-acetylcysteine levels in the supernatant significantly increased with LR growth in HBM versus formula. Lactate and succinate are involved in the glyoxylate cycle; N-acetyl cysteine is involved in methionine and cysteine metabolism and antioxidant formation.

Compared to LR 17938 cultured in formula, LR 17938 cultured in HBM produced significant changes in amino acids of different sub-pathways. We detected 261 of 452 metabolites that were up regulated, and 191 metabolites that were down regulated (*p* < 0.05) in HBM + LR compared to formula + LR. Most of the up-regulated metabolites were amino acid derivatives, and most of the down-regulated metabolites were lipids. Several metabolites that increased by greater than 5-fold in HBM + LR were involved in glycine, serine and threonine metabolism; alanine metabolism; glutamate metabolism; histidine metabolism; lysine metabolism; phenylalanine metabolism; methionine and cysteine metabolism; urea cycle; polyamine metabolism; and the glyoxylate cycle (the microbial equivalent of the tricarboxylic acid (TCA) cycle) (Table 1, Supplemental Table S1, and Figure 3A–C). 

**Figure 4:**
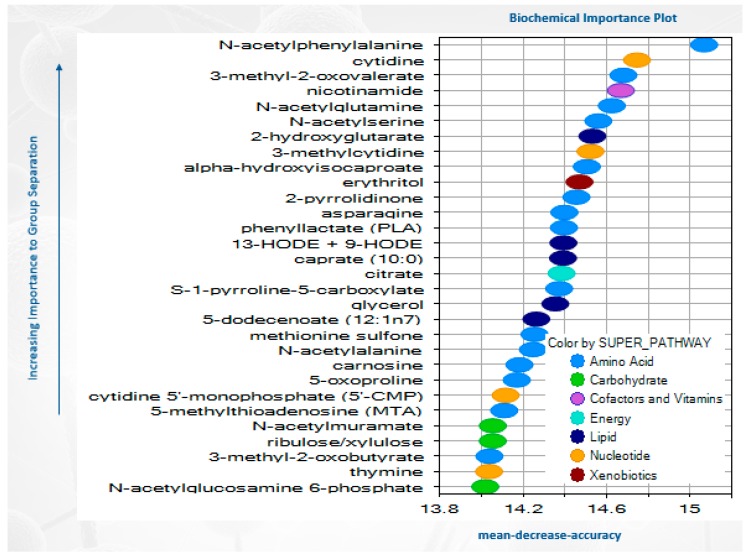
Random forest analysis showing biochemical profiles for the different samples were highly successful in binning the samples to their appropriate groups.

Random forest analysis was used to differentiate HBM with and without LR 17938, with a predictive accuracy of 100 %. The top 30 metabolites resulting from the random forest analysis in HBM seem to point heavily to changes in amino acid metabolites (including N-acetylphenylalanine, N-acetylglutamine, N-acetylserine, and N-acetylalanine) and lipid metabolites (such as caprate (10:0) and 5-dodecenoate (12:1n7)) (Figure 4).

There were 255 lipids identified from the metabolomics data. In HBM, the addition of LR 17938 caused a significant decrease in lipids, with the major involved groups including medium chain fatty acids, long chain fatty acids, polyunsaturated fatty acids, sphingomyelins (Table 2, supplemental monohydroxy fatty acids, lysophospholipids, plasmalogens, monoacylglycerols, and Table S2). Several of the decreased fatty acids with the addition of LR 17938 include linoleic acid, oleic acid, myristic acid, lauric acid, and arachidonic acid. 

### 3.3. LR 17938 Growth was Promoted by 0.05% Fatty Acids 

**Figure 5:**
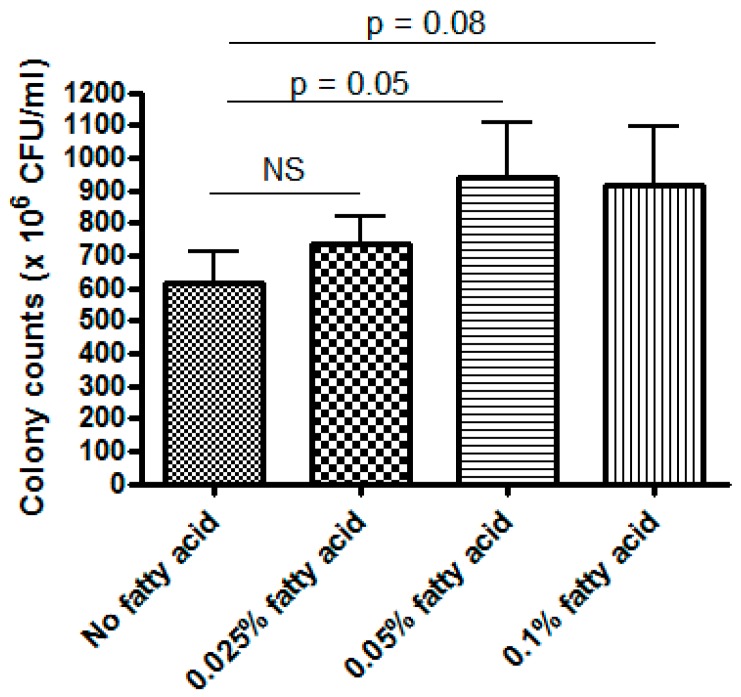
LR 17938 growth in fatty acid (0.025%, 0.05%, and 0.1%) and 20%MRS in PBS media. At 16 h, 0.05% fatty acid in MRS/PBS media promoted higher LR 17938 growth (943 ± 469 × 10^6^ CFU/mL) as compared to the MRS/PBS mixture without fatty acid (614 ± 271 × 10^6^ CFU/mL) (*p* = 0.0499). NS: no significance between group comparison (*p* > 0.05).

As LR17938 growth in HBM and HBM + CA group resulted in a significant decrease in lipids, we tested for LR 17938 growth in fatty acid supplemented culture media. While no significant changes were observed when 0.025% of fatty acids were added into 20% MRS/PBS media, 0.05% fatty acid in 20% MRS/PBS media promoted higher LR 17938 growth (943 ± 469 × 10^6^ CFU/mL) as compared to the 20% MRS/PBS without fatty acids (614 ± 271 × 10^6^ CFU/mL) (*p* = 0.0499) (Figure 5). Fatty acid (0.1% in 20% MRS/PBS media) also tended to promote higher LR 17938 growth as compared to the 20% MRS/PBS media without fatty acid, although the difference was not statistically significant (*p* = 0.08). 

**Figure 6:**
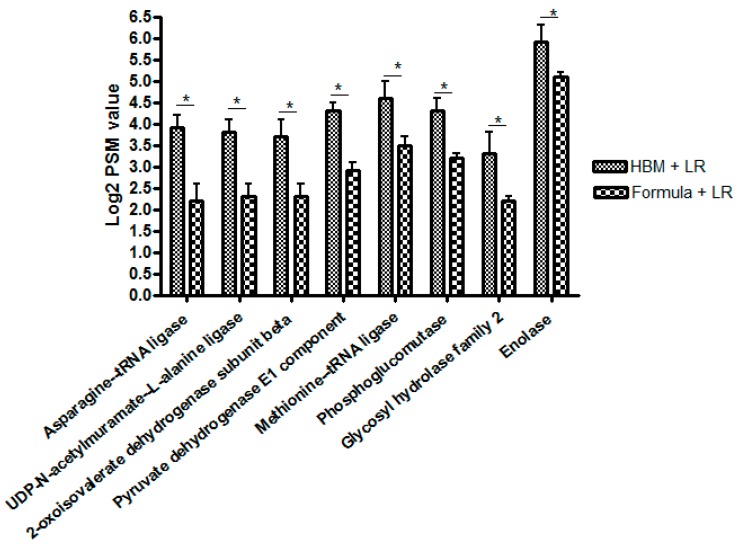
Proteomics data showing up-regulated proteins in HBM + LR group versus formula + LR group 2-oxoisovalerate dehydrogenase subunit beta, pyruvate dehydrogenase E1 component, and enolase are involved in acetyl CoA formation in glyoxylate cycle. Methionine-tRNA ligase is involved with cysteine formation (precursor to antioxidant glutathione). * All with *p* < 0.05.

Proteomic studies. Proteomics data showed 11 upregulated proteins and 19 downregulated proteins, comparing between the HBM + LR versus formula + LR groups. Among those, three upregulated proteins (pyruvate dehydrogenase E1 component, enolase, and 2-oxoisovalerate dehydrogenase subunit beta) are involved in acetyl-CoA formation in glyoxylate cycle. One upregulated protein (methionine-tRNA ligase) is involved in cysteine formation (a precursor to antioxidant glutathione) (Figure 6). 

## 4. Discussion

This is the first in vitro study looking at LR 17938 growth in human breast milk and the beneficial effect of human breast milk on microbial proliferation of LR 17938. Our study demonstrated that LR 17938 growth is significantly better in human breast milk as compared to formula (*p* < 0.001). There might be components in the human breast milk that are lacking in formula that can promote LR 17938 growth, and lipids could be a contributing factor. In fact, when we grew LR 17938 in a fatty acid mixture, 0.05% fatty acids promoted better LR 17938 growth as compared to media without fatty acid. A previous in vitro study demonstrated that Lactobacilli, when grown anaerobically in MRS medium supplemented with free polyunsaturated fatty acids (PUFA), incorporates these free PUFA into their cell lipids [21]. Translationally, a systematic review and meta-analysis of human randomized, controlled trials also showed that *in vivo*, total cholesterol and LDL-C were significantly reduced by the consumption of Lactobacilli [22]. 

LR 17938 has been previously suggested to have anti-inflammatory effects. Our metabolomics data showed several LR 17938 -associated metabolites that are specifically promoted by human breast milk that were essential to ATP production (succinate) and antioxidant or anti-inflammatory function (among those glutamine, N-acetylcysteine (NAC), citrulline, spermidine, and lactate). Glutamine, with a greater than 400-fold increase in the HBM + LR group versus formula + LR, is an amino acid which has been shown to improve gut barrier function and recovery from injury in animal models and preventive effects in experimental models of intestinal injury [23]. N-acetylcysteine, well-known for its antioxidant effect, was increased 12-fold in the HBM + LR *versus* formula + LR group. In vitro, NAC significantly reduced the expression of inflammatory markers, including tumor necrosis factor (TNF)-α, nuclear factor kappa light chain enhancer of activated B cells (NF-kB), Interferon (IFN)-γ, and interleukin (IL)-6, in lipopolysaccharides (LPS) induced IPEC J2 cells [24]. Citrulline is a marker for viable intestinal cells that protects piglet intestinal monolayer tight junctions from hypoxia-related damage [25,26]. 

In addition, we found that spermidine went up 44-fold higher in LR 17938 cultured in HBM than in formula. Spermidine belongs to the polyamine cycle, the products of which have been shown to be required for intestinal mucosal repair by modifying the expression of various growth-related genes [27]. Tofalo R has reviewed polyamines and the microbiota and shows several pathways are activated by the polyamine-associated microbiota [28]. The polyamines can be produced by probiotics including *Bifidobacterium animalis ssp. Lactis* LKM512 and *Lactobacillus rhamnosus* GG [28]. One yeast that is used as a probiotic, *Saccharomyces cerevisiae*, makes high levels of ornithine and spermidine [29,30]. This may underlie some of its reported beneficial effects in children with diarrhea. Lactate production, as demonstrated in Figure 3A, is in agreement with our reported data on LR 17938 growth in different media (Figure 1). *In vivo*, an increase in lactate concentration in colon was shown to have protective effects against 2, 4, 6–trinitrobenzenesulfonic acid (TNBS) -induced colitis [31]. N-acetylphenylalanine (a glucosamine derivative) was demonstrated in the random forest analysis to be the most importance in group separation (1-fold increase in HBM + LR/formula + LR). N-acetylphenylalanine was shown to have anti-inflammatory effects, by interfering with activation of NF-kB and activator protein (AP)-1 transcription factors and inhibiting IκB kinase (IKK)α kinase activity in vitro [32,33].

A previous in vitro study of PUFA supplementation and its effect on *lactobacillus* cell lipids suggested that the role of lactobacilli as regulators of PUFA absorption may represent another means by which probiotics can redirect the balance of inflammatory mediators derived from PUFA within the inflamed tissue [21].

Principle component analysis (PCA) from the metabolomics study showed major segregation among different media with and without LR 17938, suggesting that the metabolic differences between the groups were driven mainly by the biochemical composition of the culture media. The hierarchical clustering analysis (HCA) was more successful in clustering the samples based on LR 17938 growth status. These together suggest that the produced metabolites are from the interaction between different media and LR 17938.

Although our studies did not show a significant difference in LR 17938 growth in regular formulas versus HMO formula, a previous in vitro study did show a different metabolite production produced by the utilization of HMO (2′-fucosyllactose and 3-fucosyllactose) by probiotic bacteria [34]. Because our study focuses primarily on the difference in LR 17938 growth in HBM *versus* formula and its metabolites and proteins, and because LR 17938 growth in HMO formula was not significantly different from other formulas, we did not include HMO formula in our metabolomics and proteomics study. Metabolomics and proteomics studies of this subgroup may be considered in future studies. 

A literature search showed no previous proteomics data looking at LR 17938 growth in HBM or formula. We found that 11 proteins were upregulated and 19 proteins were downregulated when LR 17938 was cultured in HBM. Among the proteins, pyruvate dehydrogenase E1 component, enolase, and 2-oxoisovalerate dehydrogenase subunit beta were upregulated, each of which is involved in acetyl-CoA formation. This is in agreement with our metabolomics study, showing an increase in succinate, an intermediate metabolite in the glyoxylate cycle. The upregulated methionine-tRNA ligase is involved in cysteine formation. Cysteine, a product of the methionine pathway, (as mentioned) is a precursor to the antioxidant glutathione. This finding is also in agreement with our metabolomics study, which showed an increase in the antioxidant N-acetylcysteine. 

## 5. Conclusions

In summary, human breast milk enhances the growth of *Lactobacillus reuteri* DSM 17938 as compared to formula. We do not believe that the LR 17938 -altered metabolites are all simply byproducts of normal microbial growth but rather represent alterations in metabolic pathways evoked by the substrate. This, of coursem is only a speculation. We are currently looking at metabolic products of LR 17938 given to newborn mouse pups, a better model in our opinion of how the microbe interacts with luminal contents including milk source *in vivo.* In the current studies, we found significant increases in LR 17938 -associated metabolites related to ATP production and antioxidant status promoted by HBM. Our previous work linked ATP-derived adenosine/inosine to immune modulation in mice with autoimmune disease. These metabolites may be linked to the mechanism of action of *L. reuteri*. Studies are underway to determine the anti-inflammatory effects and ability to protect intestinal epithelial tight junctions of selected metabolites produced by LR 17938 in the presence of HBM.

## Figures and Tables

**Table 1:** Elevated amino acids involved in anti-inflammation function and succinate (part of glyoxylate cycle to generate ATP) in HBM + LR group as compared to formula + LR group.

Metabolite	Cycle	HBM + LR/Formula + LR
Glutamine	Glutamate Metabolism	439 fold
Spermidine	Polyamine Metabolism	44 fold
N-Acetylcysteine (NAC) (=glutathione precursor)	Methionine, Cysteine, SAM and Taurine Metabolism	12 fold
Citrulline	Urea Cycle; Arginine and Proline Metabolism	9 fold
Succinate	Glyoxylate Cycle	5 fold

**Table 2:** Decreased lipids in HBM with LR in comparison to HBM without LR.

Subpathway	Number of Molecules Involved
Sphingomyelins	16
Monoacylglycerol	15
Long Chain Fatty Acid	14
Polyunsaturated Fatty Acid (n3 and n6)	13
Lysophospholipid	13
Plasmalogen	10
Fatty Acid, Monohydroxy	7
Medium Chain Fatty Acid	6

## Supplementary Materials

The following are available online at https://www.mdpi.com/2072-6643/11/7/1548/s1, Figure S1: LR growth in HBM alone *versus* HBM + 1% CA. 1% CA, when supplemented to HBM, promoted LR growth more than three times as compared to LR growth in HBM alone (*p* < 0.001). Figure S2: LR growth in regular formulas *versus* HMO formula, without casamino acid (left) and with casamino acid (right). LR growth was not significantly better in HMO formula *versus* regular formulas. Table S1: Elevated metabolites in HBM + LR *versus* formula + LR group, with 5 fold increase or higher, Table S2: Decreased lipids in HBM with LR in comparison with HBM without LR group.
