# Supplementary material for: Human Breast Milk Promotes the Secretion of Potentially Beneficial Metabolites by Probiotic Lactobacillus reuteri DSM 17938
Source: Nutrients. 2019 Jul 9;11(7):1548. doi: 10.3390/nu11071548 (PMC6683045; doi:10.3390/nu11071548)
Supplement: Supplementary file 1 [file nutrients-11-01548-s001.pdf]

Supplementary Materials:

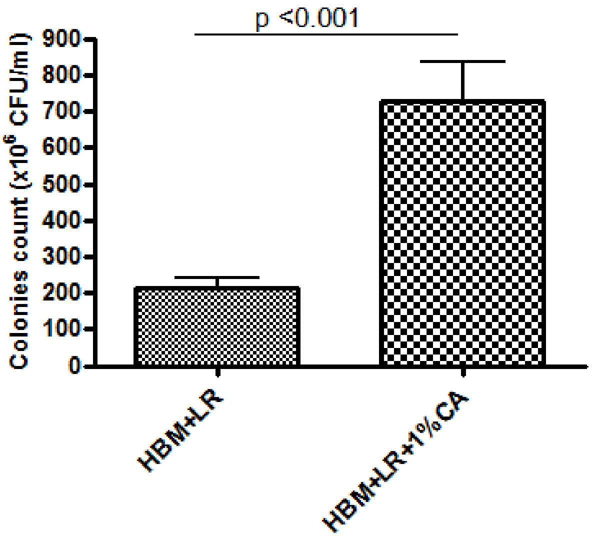

Figure S1. LR growth in HBM alone versus HBM + 1% CA. 1% CA, when supplemented to HBM, promoted LR growth more than three times as compared to LR growth in HBM alone ($p < 0.001$).

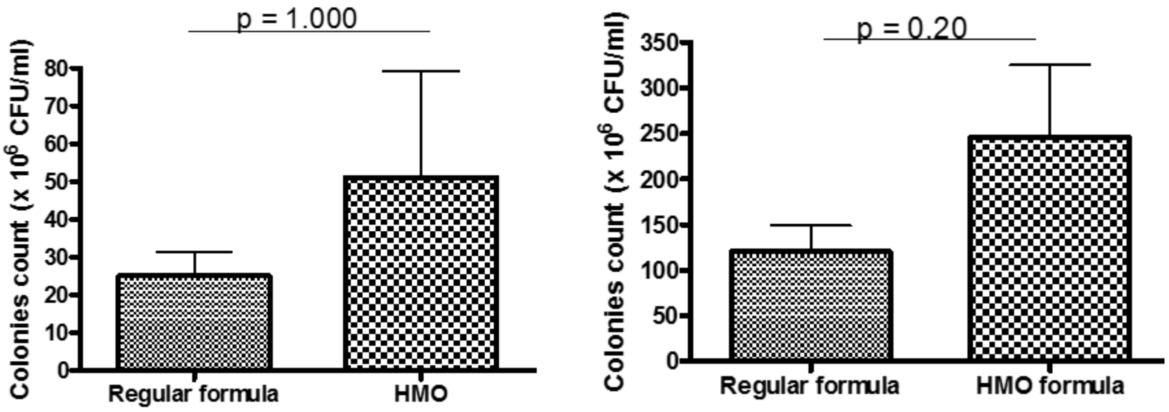

Figure S2. LR growth in regular formulas versus HMO formula, without casamino acid (left) and with casamino acid (right). LR growth was not significantly better in HMO formula versus regular formulas.

Table S1. elevated metabolites in HBM+LR versus formula + LR group, with 5 fold increase or higher.

Cycle	Metabolite	HBM + LR/ Formula + LR
Glycine, Serine and Threonine Metabolism	Glycine	51.42
	Dimethylglycine	26.95
	Serine	17.13
	Threonine	10.00
	Allo-threonine	64.78
Alanine and Aspartate Metabolism	Alanine	8.15
Glutamate Metabolism	Glutamine	439.20
Histidine Metabolism	Trans-urocanate	41.96
	Cis-urocanate	7.02
	Anserine	9.35
Lysine Metabolism	Lysine	14.65
Phenylalanine Metabolism	Phenylalanine	6.17
Methionine, Cysteine, SAM and Taurine Metabolism	N-acetylcysteine	12.51
	Cystine	21.67

Urea cycle; Arginien and Proline Metabolism	Citrulline	9.76
Polyamine Metabolism	Spermidine	44.36
	5-methylthioadenosine (MTA)	30.47
Glyoxylate Cycle	Succinate	5.25

Table S2. Decreased lipids in HBM with LR in comparison with HBM without LR group.

Cycle	Metabolite	HBM + LR/ HBM - LR
Medium Chain Fatty Acid	caproate (6:0)	0.10
	heptanoate (7:0)	0.04
	caprylate (8:0)	0.02
	caprate (10:0)	0.04
	laurate (12:0)	0.05
	5-dodecenoate (12:1n7)	0.08
Long Chain Fatty Acid	myristate (14:0)	0.05
	myristoleate (14:1n5)	0.13
	palmitate (16:0)	0.10
	palmitoleate (16:1n7)	0.06
	margarate (17:0)	0.14
	10-heptadecenoate (17:1n7)	0.07
	stearate (18:0)	0.17
	oleate/vaccenate (18:1)	0.10
	nonadecanoate (19:0)	0.24
	10-nonadecenoate (19:1n9)	0.08
	arachidate (20:0)	0.22
	eicosenoate (20:1)	0.08
	erucate (22:1n9)	0.08
	nervonate (24:1n9)*	0.08
Polyunsaturated Fatty Acid (n3 and n6)	hexadecatrienoate (16:3n3)	0.17
	stearidonate (18:4n3)	0.10
	eicosapentaenoate (EPA; 20:5n3)	0.05
	docosapentaenoate (n3 DPA; 22:5n3)	0.08
	docosahexaenoate (DHA; 22:6n3)	0.05
	docosatrienoate (22:3n3)	0.20
	linoleate (18:2n6)	0.07
	linolenate [alpha or gamma; (18:3n3 or 6)]	0.06
	dihomo-linolenate (20:3n3 or n6)	0.07
	arachidonate (20:4n6)	0.05
	docosapentaenoate (n6 DPA; 22:5n6)	0.07
	docosadienoate (22:2n6)	0.06
	dihomo-linoleate (20:2n6)	0.07
Fatty Acid, Monohydroxy	2-hydroxyoctanoate	0.73
	2-hydroxydecanoate	0.45
	3-hydroxylaurate	0.24
	3-hydroxymyristate	0.16
	3-hydroxypalmitate	0.10
	3-hydroxyoleate*	0.24
	13-HODE + 9-HODE	0.13
Lysophospholipid	1-palmitoyl-GPC (16:0)	0.11
	2-palmitoyl-GPC (16:0)*	0.28
	1-linoleoyl-GPC (18:2)	0.11
	1-palmitoyl-GPE (16:0)	0.27
	2-stearoyl-GPE (18:0)*	0.30
	1-oleoyl-GPE (18:1)	0.15
	1-linoleoyl-GPE (18:2)*	0.14
	1-arachidonoyl-GPE (20:4n6)*	0.18
	1-stearoyl-GPS (18:0)*	0.24
	1-linoleoyl-GPS (18:2)*	0.09
	1-palmitoyl-GPI (16:0)	0.30
	1-stearoyl-GPI (18:0)	0.25
	1-linoleoyl-GPI (18:2)*	0.15
Plasmalogen	1-(1-enyl-palmitoyl)-2-oleoyl-GPE (P-16:0/18:1)*	0.60
	1-(1-enyl-palmitoyl)-2-linoleoyl-GPE (P-16:0/18:2)*	0.58

	1-(1-enyl-palmitoyl)-2-palmitoyl-GPC (P-16:0/16:0)*	**0.49**
	1-(1-enyl-palmitoyl)-2-arachidonoyl-GPE (P-16:0/20:4)*	**0.62**
	1-(1-enyl-palmitoyl)-2-oleoyl-GPC (P-16:0/18:1)*	**0.39**
	1-(1-enyl-stearoyl)-2-oleoyl-GPE (P-18:0/18:1)	**0.59**
	1-(1-enyl-stearoyl)-2-linoleoyl-GPE (P-18:0/18:2)*	**0.59**
	1-(1-enyl-palmitoyl)-2-arachidonoyl-GPC (P-16:0/20:4)*	**0.43**
	1-(1-enyl-palmitoyl)-2-linoleoyl-GPC (P-16:0/18:2)*	**0.44**
	1-(1-enyl-stearoyl)-2-arachidonoyl-GPE (P-18:0/20:4)*	**0.60**
Monoacylglycerol	1-myristoylglycerol (14:0)	**0.04**
	1-pentadecanoylglycerol (15:0)	**0.07**
	1-palmitoylglycerol (16:0)	**0.07**
	1-palmitoleoylglycerol (16:1)*	**0.04**
	1-margaroylglycerol (17:0)	**0.06**
	1-oleoylglycerol (18:1)	**0.10**
	1-linoleoylglycerol (18:2)	**0.06**
	1-docosahexaenoylglycerol (22:6)	**0.06**
	2-myristoylglycerol (14:0)	**0.05**
	2-palmitoylglycerol (16:0)	**0.10**
	2-palmitoleoylglycerol (16:1)*	**0.08**
	2-oleoylglycerol (18:1)	**0.07**
	2-linoleoylglycerol (18:2)	**0.09**
	2-docosahexaenoylglycerol (22:6)*	**0.29**
	1-heptadecenoylglycerol (17:1)*	**0.23**
Sphingomyelins	palmitoyl sphingomyelin (d18:1/16:0)	**0.45**
	stearoyl sphingomyelin (d18:1/18:0)	**0.48**
	tricosanoyl sphingomyelin (d18:1/23:0)*	**0.57**
	lignoceroyl sphingomyelin (d18:1/24:0)	**0.61**
	sphingomyelin (d18:1/14:0, d16:1/16:0)*	**0.37**
	sphingomyelin (d17:1/16:0, d18:1/15:0, d16:1/17:0)*	**0.40**
	sphingomyelin (d18:2/16:0, d18:1/16:1)*	**0.29**
	sphingomyelin (d18:1/17:0, d17:1/18:0, d19:1/16:0)	**0.47**
	sphingomyelin (d18:1/18:1, d18:2/18:0)	**0.39**
	sphingomyelin (d18:1/20:1, d18:2/20:0)*	**0.37**
	sphingomyelin (d18:1/21:0, d17:1/22:0, d16:1/23:0)*	**0.47**
	sphingomyelin (d18:1/22:1, d18:2/22:0, d16:1/24:1)*	**0.44**
	sphingomyelin (d18:2/23:0, d18:1/23:1, d17:1/24:1)*	**0.44**
	sphingomyelin (d18:1/24:1, d18:2/24:0)*	**0.48**
	sphingomyelin (d18:2/24:1, d18:1/24:2)*	**0.45**
	sphingomyelin (d18:1/25:0, d19:0/24:1, d20:1/23:0, d19:1/24:0)*	**0.40**
